# Legacy of contaminant N sources to the NO_3_^−^ signature in rivers: a combined isotopic (δ^15^N-NO_3_^−^, δ^18^O-NO_3_^−^, δ^11^B) and microbiological investigation

**DOI:** 10.1038/srep41703

**Published:** 2017-02-02

**Authors:** Cyrielle Briand, Mathieu Sebilo, Pascale Louvat, Thierry Chesnot, Véronique Vaury, Maude Schneider, Valérie Plagnes

**Affiliations:** 1 UPMC Univ Paris 06, UMR IEES, 4 place Jussieu, 75252 Paris Cedex 05, France; 2 Institut de Physique du Globe de Paris, Sorbonne Paris Cité, Université Paris-Diderot, UMR CNRS 7154, 1 rue Jussieu, 75238 Paris Cedex, France; 3 Eurofins Expertises Environnementales, Microbiologie, Santé-Environnement, rue Lucien Cuenot/site St Jacques II, 54521 Maxeville, France; 4 UPMC Univ Paris 06, UMR METIS, 4 place Jussieu, 75252 Paris Cedex 05, France

## Abstract

Nitrate content of surface waters results from complex mixing of multiple sources, whose signatures can be modified through N reactions occurring within the different compartments of the whole catchment. Despite this complexity, the determination of nitrate origin is the first and crucial step for water resource preservation. Here, for the first time, we combined at the catchment scale stable isotopic tracers (δ^15^N and δ^18^O of nitrate and δ^11^B) and fecal indicators to trace nitrate sources and pathways to the stream. We tested this approach on two rivers in an agricultural region of SW France. Boron isotopic ratios evidenced inflow from anthropogenic waters, microbiological markers revealed organic contaminations from both human and animal wastes. Nitrate δ^15N^ and δ^18^O traced inputs from the surface leaching during high flow events and from the subsurface drainage in base flow regime. They also showed that denitrification occurred within the soils before reaching the rivers. Furthermore, this study highlighted the determinant role of the soil compartment in nitrate formation and recycling with important spatial heterogeneity and temporal variability.

High nitrate concentrations in surface and ground waters remain a worldwide concern because of sanitary problems in drinking water and of ecological disturbances in aquatic systems such as eutrophication^1^,^2^,^3^. Hence, the determination of nitrate origin is the first step for effective management plans aiming at preserving surface water quality. Nitrate concentrations in rivers are controlled by spatial and temporal variability of the different nitrate sources and biogeochemical or physical reactions occurring from uplands to streams^4^,^5^,^6^ (Fig. 1). The nitrogen and oxygen isotope ratios of NO_3_^−^ (δ^15^N and δ^18^O) have been widely used to investigate the sources of NO_3_^−^ in rivers and groundwater^4^,^6^,^7^,^8^,^9^,^10^,^11^,^12^,^13^,^14^. Actually, at the catchment scale, some N sources, such as domestic and animal effluents, present overlapping δ^15^N ranges. The isotopic signature of nitrate results also from processes within the soils that modify its concentration (nitrification, denitrification, Fig. 1)^15^,^16^,^17^ fractionate its isotopes, and can blur the initial signature of the N sources. These limits can be over passed by using complementary tracers.

Previous studies have investigated boron isotope ratio (δ^11^B) to better segregate among the different nitrate sources^18^,^19^,^20^,^21^,^22^,^23^. In most of the NO_3_^−^ contamination sources, B is also substantially enriched^18^ and presents the advantage of not being affected by oxidation/reduction and biological reactions involving N compounds. δ^11^B has been particularly effective at distinguishing domestic from animal effluents^18^,^19^,^20^,^21^,^22^,^23^. However, B adsorption on solid surfaces such as soil particles significantly fractionates its isotopes, possibly limiting its use as tracer of sources^18^,^24^,^25^,^26^,^27^.

In parallel, researchers have developed microbiological markers able to distinguish animal and human fecal contaminations^28^,^29^,^30^. F-specific RNA phages (FRNAPHs) are viruses infecting *Escherichia Coli* and can be divided into four groups (G), with GI and GIV mostly associated with animal feces and GII and GIII generally characteristic of human contamination^31^,^32^,^33^. Bacteroidales are fecal bacteria with DNA sequences specific to human, cattle, pig effluents^34^,^35^,^36^,^37^. These host-specificity markers have rarely been applied in natural aqueous environments^38^,^39^, but their recent combination with δ^15^N, δ^18^O and δ^11^B has shown a great potential for determining NO_3_^−^ origin in groundwater (GW)^38^.

The objective of the present study was to determine the origin of nitrate in rivers where multiple nitrogen sources co-exist (Fig. 1). To do so, isotopic (δ^15^N, δ^18^O and δ^11^B) and microbiological markers were combined for the first time at the watershed scale. This multi tracers approach was tested on two rivers of the southwest of France in an agricultural region (Fig. 2). Nitrate concentrations of Gabas River (GR, 150 km long) and Laudon River (LR, 15 km long) are a serious threat for the underlying karstic aquifer, used for drinking water supply^38^. To better integrate the spatial heterogeneity of N point and non-point sources of the whole watershed, a 2.5 years monitoring has been conducted in the karstified downstream part of the Gabas and Laudon catchments (Fig. 2). Water sampling was carried out every two months in base flow conditions but also during flood events (Fig. 3) to characterize NO_3_^−^ dynamics in rivers. Additionally, samples of rain, waste water treatment plant (WWTP) effluents, manure and animal fresh dejections were collected to determine the isotopic and microbiological signature of the local N sources (Fig. 2). Finally, samples of agricultural soil, water from buried drains and surface ditch were sampled to follow the N fluxes from topsoil to rivers (Fig. 2).

## Results and Discussion

Results for each tracer are first presented and discussed separately in order to understand how they can trace the different water sources but also to highlight their limits. Then we discuss how the tracer’s combination allows a global understanding of NO_3_^−^ sources, reactions and pathways.

### Nitrate concentrations and stable isotopic compositions (δ^18^O and δ^15^N): source mixing and N cycling within the soils

Nitrate concentrations [NO_3_^−^] in Gabas River (GR) and Laudon River (LR) both ranged from 12 to 45 mg.L^−1^ (Table 1). They were globally equals to [NO_3_^−^] of the water from surface ditch (n = 6) but mostly inferior to [NO_3_^−^] of WWTP effluents (n = 3) and buried drains (n = 3) (Table 1, Fig. 4). GR and LR nitrate also exhibit similar ranges of δ^15^N (+8.0‰ to +14.6‰) and δ^18^O (+3.8‰ to +9.3‰, Table 1, Fig. 5) that are usually encountered for nitrate derived from organic N sources such as manure and sewage. NO_3_^−^ extracted from topsoil samples showed the lowest δ^15^N signatures (−11.8‰ to +4.6‰) with a large range of δ^18^O (+5.5‰ to +11.4‰, Table 2, Fig. 5), whereas buried drains exhibit the highest δ^15^N (+11.4‰ to +19.6‰) and δ^18^O (+7.3‰ to +11.0‰, Table 1, Fig. 5).

**Figure 1:**
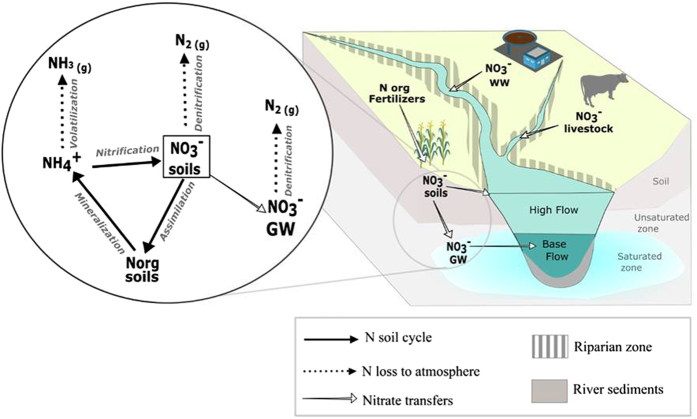
Schematic river catchment and its sources of nitrogen. WW = waste waters, GW = groundwater. (Created with Inkscape, version 2, https://inkscape.org/fr/, *images from Courtesy of the Integration and Application Network, University of Maryland Center for Environmental Science* (ian.umces.edu/symbols/)).

The large gap between δ^15^N and δ^18^O of nitrate extracted from topsoil and nitrate of the drainage network (ditches and buried drains) reflects that important processes occur within the soils before reaching the studied rivers. *It has recently been shown that residual fraction of NO*_*3*_^*−*^ *that is not immediately leached or consumed by plants is assimilated into soil organic matter and potentially recycled into NO*_*3*_^*−*^ 40 (Fig. 1*). Some of the reactions involved in the N cycle, such as NH*_*4*_^+^ *volatilization and NH*_*4*_^+^ *nitrification are important isotope fractionating processes*^16^,^17^,^41^,^42^*. N availability and reaction rate of each process will thus control the large δ*^*15*^*N range of newly produced nitrate*^16^,^43^*. Additionally, in top-soils, water evaporation and the subsequent increase of δ*^*18*^*O-H*_*2*_*O associated to root penetration will potentially lead to higher δ*^*18*^*O-NO*_*3*_^*−*^44*, as 2 atoms of oxygen in NO*_*3*_^*−*^ *come from the surrounding water*^7^,^16^,^45^,^46^*. This explains why the highest δ*^*18*^*O of soil extracted NO*_*3*_^*−*^ *was measured in august 2012, under mature maize and when potential evaporation was maximum, and the lowest δ*^*18*^*O-NO*_*3*_^*−*^ *in January in absence of vegetal cover and at low temperature. Consequently, N cycling and the physical processes occurring within the soil compartment result in important temporal variations of δ*^*15*^*N* *and δ*^*18*^*O of the produced NO*_*3*_^*−*^*. These observations highlight the determinant role of the water transfer through soils over the nitrate isotopic signature, and alter the initially distinct δ*^*15*^*N* *(δ*^*15*^*N*_*urea*_* *=* 0.9‰, δ*^*15*^*N*_*manure*_* *=* 9.3‰), and δ*^*18*^*O (NO*_*3*_^*−*^ *fertilizers vs. atmospheric deposition) of the N source end-members.*

If no trend can be observed between [NO_3_^−^] and rivers flow rates (Fig. 6), for both rivers, the lowest δ^15^N and δ^18^O values were mainly observed at high water stage (>2 m^3^.s^−1^ for GR) whereas the highest were measured for base flow (<2 m^3^.s^−1^ for GR, Fig. 6). These higher δ^15^N values could be due to a larger contribution of an enriched pool of nitrate such as WWTP effluents (δ^15^N = +10.0‰ to +17.3‰) (Figs 4 and 5) during base flow compared to high flow events. However, the δ^18^O of NO_3_^−^ in the WWTP outlet (+6.4‰ to +8.5‰) are lower than the maximum values measured in GR and LR and thus cannot explain the δ^18^O enrichment also observed for base flow samples (Fig. 5). Above all, considering the high [NO_3_^−^] of WWTP effluents, a larger contribution of this of this pool should also have increased [NO_3_^−^] in rivers, which is not observed (Fig. 4). A simple mixing of N sources thus cannot explain these high nitrate δ^15^N and δ^18^O during base flow and additional processes must be involved. Actually the nitrate δ^15^N and δ^18^O of LR samples, are pretty well distributed along the 2:1 slope expected for residual nitrate derived from denitrification^15^,^16^,^47^ (Fig. 5) and are roughly inversely correlated with [NO_3_^−^] (Fig. 4). For the GR samples, δ^15^N and δ^18^O values do not strongly follow the denitrification slope but a global positive trend exists between high- and base-flow samples. Chen *et al*.^6^, reported a similar pattern with seasonal distribution in the Beijang catchment and concluded that denitrification occurred within the watershed soils, before residual NO_3_^−^ reached the river^6^,^7^.

For the samples collected under high flow conditions (Fig. 6), nitrate δ^15^N vary largely while δ^18^O remain quite constant, which excludes denitrification processes. In this case, the low δ^15^N compared to base flow samples might be explained by a larger contribution of a ^15^N depleted nitrate pool. The first possibility is that during these highly rain periods, the atmospheric nitrate (δ^15^N = +1.6‰) decreases the river δ^15^N-NO_3_^−^ signal. However, if such mixing process had occurred, it would also have increased the δ^18^O-NO_3_^−^ of rivers, which is not observed here (Fig. 6). But above all, the very low [NO_3_^−^] of rain (2.7 mg.L^−1^, Table 1) compared to rivers [NO_3_^−^] during flood events (globally superior to 20 mg.L^−1^, Table 1) seems unlikely to have impacted the global δ^15^N-NO_3_^−^ of rivers. The second possibility is that runoff over saturated soils brings topsoil NO_3_^−^, characterized by low δ^15^N (−11.8 to +4.2‰), to rivers and globally decreases the river δ^15^N-NO_3_^−^.

### Boron δ^11^B ratios: natural versus anthropogenic signatures

Boron analyses have been carried out on GR and LR samples on the base of nitrate results, to best represent the largest variability of [NO_3_^−^], δ^15^N and δ^18^O. GR, LR and drainage waters, except two drain samples, were characterized by low boron concentrations (1.8 to 14.9 μg.L^−1^, Table 1, Fig. 7), usually encountered in uncontaminated water^19^,^48^. The δ^11^B of these samples (+13.5‰ and +26.8‰) plots into the overlapping typical ranges of rain^40^ and manure sources^19^,^42^ (Fig. 7). The δ^11^B measured for local rain (27.7‰) and WWTP effluents (1.9‰) were in good agreement with the ranges reported in literature for atmospheric and domestic boron^19^,^22^ (Fig. 7). However, the characterization of local animal pool (fresh dejection δ^11^B = −3.3‰ and poultry manure δ^11^B = +8.6‰) shows lower values than previously reported (+15.3‰ to +27.6)^19^. Globally, [B] and δ^11^B of the GR and LR samples were really close to those measured in the underlying karstic aquifer (10.5 ± 2 ppb and 25.3 ± 1‰)^38^, and in rain, and were significantly different from the WWTP end-member (Fig. 7).

The isotopic shift between the measured δ^11^B values and those usually referenced for the animal pool may be due to the different experimental procedures: total digestion by alkaline fuse for our measurements versus leaching for the literature values. The ^10^B has a greater affinity for sorption onto solid surfaces^24^,^25^,^27^ than ^11^B, and leaching experiments might have induced an isotopic fractionation towards higher δ^11^B in the leach solutions compared to the bulk values from alkaline fusion. The measured δ^11^B signatures of the animal end-member plot close to the domestic (WWTP) pool, making δ^11^B not effective to distinguish animal from human contamination. Drain 2 exhibits higher [B] and lower δ^11^B than the other surface water samples and could reflect a more important contribution of animal or domestic contamination.

### Microbiological markers: animal versus domestic contamination

Human *Bacteroidales* were encountered at significant concentrations (>10^4^ copies/100 mL) in WWTP effluents (n = 1), in the 4 ditch samples and in GR (10 times among 11 analyzed samples, or 10/11) and LR (11/15) under both base and high-flow rates (Table 1). This domestic fecal contamination was corroborated by the detection of FRNAPHs of Group II at significant concentrations (proportion greater than 20% of total phages counted on more than 12 phages) in two of the 4 ditch samples, in 2 high flow LR samples and in both high- (n = 3) and base-flow (n = 2) GR samples (Table 1). These results are consistent with the fact that domestic rejects are constant and do not depend on hydrology or season. Animal contamination was pointed out in both rivers. Among the eleven samples from GR, cattle and pig markers were detected in respectively 7 and 4 samples, mostly collected under high flow conditions. Among the 15 samples of LR, cattle Bacteroidales were detected ten times in both high (6/10) and base flow samples (4/10) while pig markers were only detected in high flow samples (5/15). Finally, duck-chicken-goose marker was tested during the flood event of the last campaign and was detected in all GR and LR samples. These results reflect that runoff is the major pathway of microorganisms from animal sources to rivers through surface leaching during rainy events, but the markers do not allow to distinguish contamination from point sources, such as fresh dejections in farms, and non-point sources, such as manure spread on fields. However, as no animal marker was found (at significant concentration) in the ditches that drain maize fields, it could traduce that animal contamination measured in GR and LR rather arises from farming rather than from manure spread on fields. For LR, the regular presence of cows in the Laudon 200m upstream the sampling point can explain how cattle markers can reach the LR in absence of rain event.

The microbiological tracers undeniably indicate that domestic and animal fecal contaminations do impact the two rivers and thus can potentially contribute to NO_3_^−^ contents of GR and LR.

### Processes and pathways within catchment

In this study, anthropogenic contaminations have been evidenced by δ^11^B in the LR and GR rivers (without possible distinction between WWTP and animal sources), and both animal and human microbiological markers have been detected. Because the identified sources of microbiological tracers and of boron also contain high N levels, their combination with the δ^15^N and δ^18^O of nitrates offers a better understanding of the processes and pathways of nitrates to the rivers as the δ^11^B signatures of the rain and GW are significantly different from those of animal and domestic sources, they can be used for tracing and quantifying the proportions of B arising from these different groups of B sources. A simple mixing calculation (details in Methods) based on δ^11^B and [B] of three potential B sources (karstic groundwater, WWTP effluents and rainwater) was applied to each river sample. Results indicate that the atmospheric pool is the main source of boron to the rivers (50 to 96%), while groundwater brings a smaller proportion of boron (4 to 45%) and that domestic effluents (WWTP) contribute to a maximum of 10% of the total river boron (0 to 5% for GR and 0 to 10% for LR). This simplified model does not integrate the animal end-member because alkaline fusion procedure did not allow to characterize [B] arising from animal manure and fresh dejection leaching. However, considering the overlapping δ^11^B signatures of the animal and domestic effluents, it comes that at least a part of the calculated WWTP contribution could actually be of animal origin. Alternatively, the low [B] and high δ^11^B measured in GR and LR could also result from B adsorption onto soils particles before boron reached rivers with an isotopic ^11^B enrichment^20^,^39^,^50^. Such a process could have blurred a larger contribution of domestic and/or animal effluents than previously deduced through the mixing calculation. B adsorption is more prone to occur within soils and GW (longer water residence time and higher rock/water ratio) than during surface leaching or within the rivers, and it is already expressed in the δ^11^B signatures of the GW and drainage samples (drains and ditches). The similar B signatures of GW and rain can explain the high constancy of [B] and δ^11^B measured in the two rivers during base flow, when GW is the major water source, as well as during high flow episodes, when rain contribution strongly increases. If microbiological tracers have shown that domestic and WWTP effluents are permanent contaminations for GR and LR, the denitrification signal identified by δ^15^N and δ^18^O of the GR and LR nitrates for base flow samples only associated with constant δ^11^B should rather reflect changes of NO_3_^−^ pathways than changes of NO_3_^−^ sources. During base flow regime, soils are not saturated, rain or irrigation water infiltrate within the soils carrying dissolved nitrate to the saturated zone where chemical and physical conditions (temperature, soil humidity, dissolved oxygen concentrations…) and agricultural practices (N-fertilizer input periods) control the degree of denitrification. In absence of surface runoff, this shallow GW is the major source of water to GR and LR, bringing denitrified nitrate to rivers. This hypothesis is comforted by the high [NO_3_^−^], δ^15^N and δ^18^O measured in the buried drains (Figs 4 and 5) but also by the close boron signatures of drain 1, LR and GR samples. On the contrary, when the soils of the catchment are saturated, runoff increases river’s flows and surface leaching. Topsoil nitrates, that have not yet undergone denitrification, thus become an additional NO_3_^−^ source for rivers. These nitrates arise, at least partially, from animal sources, as was deduced from the detection of cattle microbiological markers.

## Conclusion

Isotopic and microbiological tracers have proven very powerful for the determination and characterization of contaminant sources to rivers. However, physical and chemical reactions of the N cycle within the soils blur the initial N signatures and soils become an additional source of newly produced (or transformed) nitrate, which contribution to the river depends on the hydrological stage of the catchment. That’s why combining of these different tracers associated to temporal monitoring are required to explain the variations of nitrate concentrations and isotopic signatures measured in the two rivers. In the present agricultural catchment with nitrate pollution threat to the underlying karstic aquifer, anthropogenic contaminations were identified through *δ*^11^B measurements, microbiological tracers recorded animal dejections during high-flow stages but permanent human effluents. *δ*^*15*^*N and δ*^*18*^*O* of nitrate allowed to understand the N-cycle within the soils and its impact on the nitrate pathways to the rivers. It thus appears essential to monitor at least as much as for rivers themselves, the spatial heterogeneity and the temporal variability of nitrate concentrations and isotopic compositions in topsoil but also in drains and ditches in order to characterize the contribution of soil to the global nitrate content of rivers. This study also highlighted the crucial impact of hydrological conditions on nitrate contents and signature in rivers.

## Methods

### Study site

**Figure 2:**
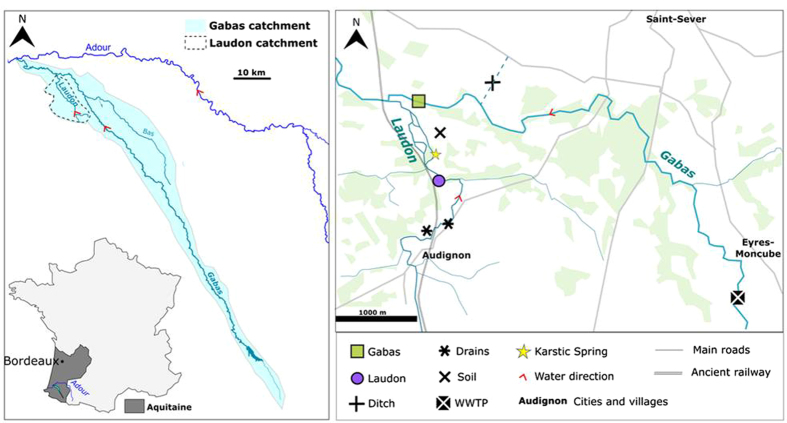
Left: Location of the Gabas and Laudon catchments in the southwest of France. Right: Zoom of the study area with the location of sampling points. *(Created with Inkscape, version 2,* https://inkscape.org/fr/).

**Figure 3:**
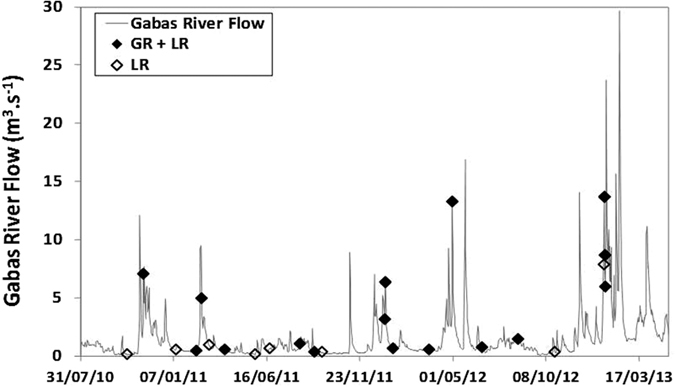
Timing of sampling campaigns on Gabas River and Laudon River versus the Gabas River flow rate series.

**Figure 4:**
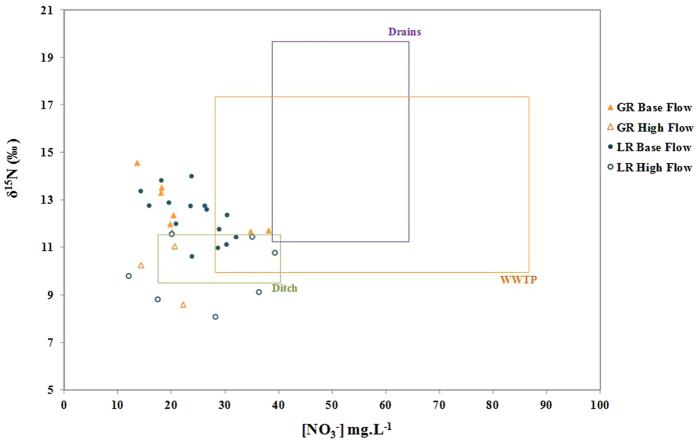
δ^15^N versus nitrate concentrations measured in the Gabas River (GR) and the Laudon River (LR) during base flow and high flow regimes. Ranges measured in surface ditch, buried drains, soil extractions and wastewater plants (WWTP) effluents are reported.

**Figure 5:**
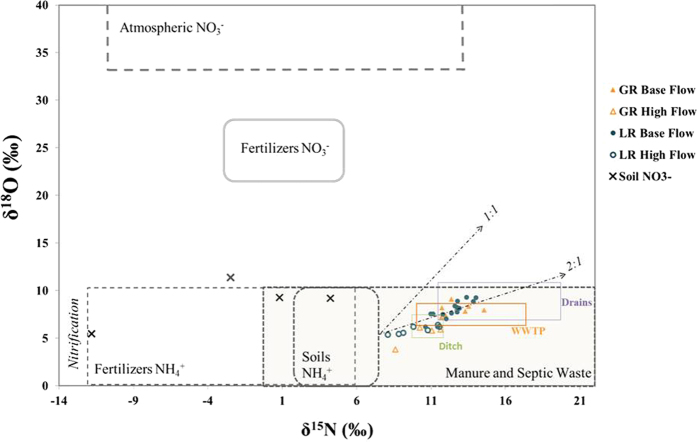
δ^18^O and δ^15^N of nitrate measured in Gabas River (GR), Laudon River (LR) during base flow and high flow. Ranges measured in surface ditch, buried drains, soil extractions and wastewater plants (WWTP) effluents are presented as well as the typical ranges of the different nitrate end-members^16^ and the two typical trends (1:1 and 2:1) observed in literature for denitrification.

**Figure 6:**
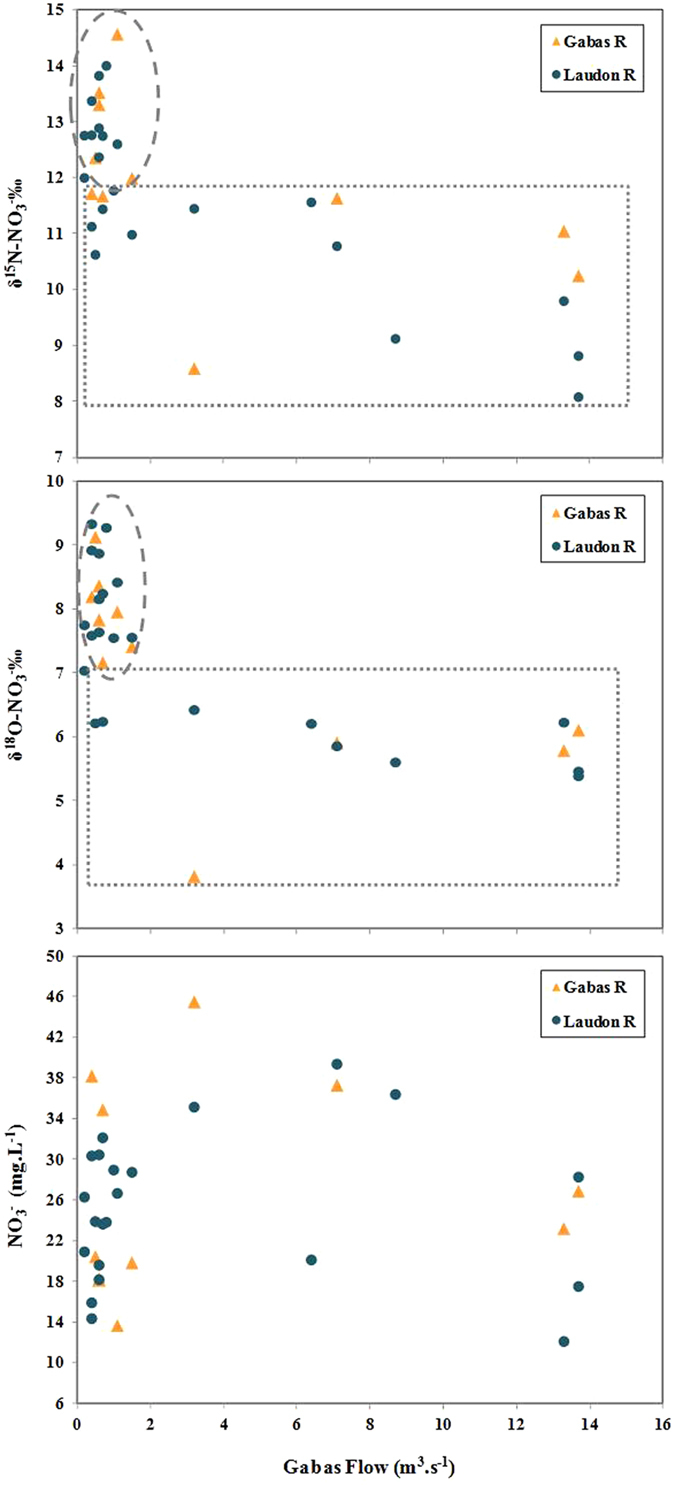
Nitrate concentrations, δ^15^N and δ^18^O depending on the Gabas River flow (m^3^.s^−1^) measured in the middle part of the Gabas section.

**Figure 7:**
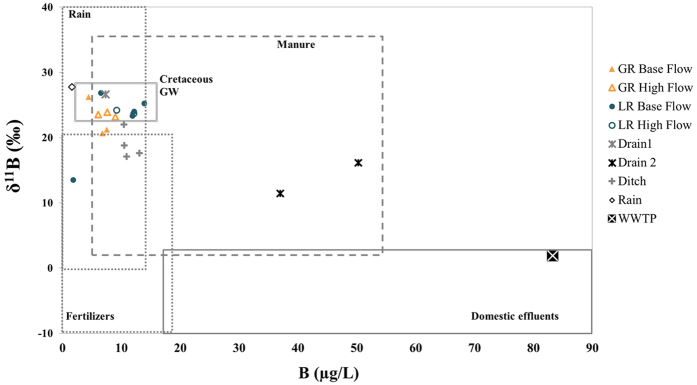
δ^11^B versus concentration of boron in surface water (Gabas River, Laudon River, drains, ditch), WWTP effluents and rain. Expected ranges reported in literature for boron end members are also presented^19^,^38^,^48^,^49^,^50^.

Located in the southwest of France, Gabas River and its tributary Laudon River, respectively drain catchment areas of 420 km^2^ and 50 km^2^ 51 (Fig. 2). In the upstream part of its catchment, Gabas dug into sandy-clay molasses of Eo-Miocene. In the downstream part, Gabas and Laudon incise Cretaceous and Eocene karstic carbonate formations (anticline structure). Reliefs correspond to Miocene sandy formation and represent potential perched aquifers of little extension^51^. Gabas catchment and the sub-catchment of Laudon are mainly devoted to agriculture (80% of the total surface) with intensive maize cultivation and farms, evolving from cattle and pigs in the upstream part to poultry in the downstream part^51^. The two rivers are largely used for maize irrigation from April-May to August-September, depending on spring and summer rainfalls. Potential local sources of nitrate are thus, urea and manure applied on maize fields, nitrification of soil organic matter, livestock slurry and domestic effluents. The high [NO_3_^−^] regularly measured in GR and LR, close to the European drinking limit of 50 mgNO_3_^−^.L^−1^ (European Directive 98/83/CE), represents a serious threat for the underlying karstic aquifer which is a strategic resource of drinking water^51^. Base flow of Gabas is around 1 to 3 m^3^/s (Fig. 3) and if it is globally inferior for Laudon with 0.5 m^3^/s, the variations of water levels are synchronous between the two streams. Flood responses are very rapid (a few hours), with maximum flows above 10 m^3^/s and 1 m^3^/s respectively for GR and LR.

### Sampling strategy

Rivers water sampling has been carried out from October 2010 to January 2013. Sampling was realized at the very output of each watershed (Fig. 2) to better integrate the different point and non-point sources of nitrate occurring in the whole catchments and that potentially infiltrate to groundwater through the karstic outcrops. As agricultural activities evolve within the year whereas domestic inputs are more constant, sampling has been realized at different step of agricultural practices: before and after N inputs, under and without maize cover, in order to follow potential changes in agricultural/domestic contributions. Moreover, because biogeochemical processes affecting nitrate depend on parameters such as meteorology and hydrology, samples have been collected under different hydrological conditions: during base flow regimes and flood events (Fig. 3).

LR and GR were sampled 1 km before their confluence (Fig. 2). The lack of automatic measurements of LR flow rates forced us to use the Gabas chronicles for the interpretation of the LR data.

The different local potential sources of nitrate were collected to characterize their isotopic signature. Solid samples of fertilizers (urea pellets), cattle manure and fresh dejections (ducks) were provided by local farmers. Wastewater treatment plant effluents were sampled three times before its discharge in GR (Fig. 2). Rainwater samples have been collected in the downstream part of the GR catchment, less than 1 km from both river monitoring points (Fig. 2). An agricultural soil (maize field) located in the Laudon catchment (Fig. 2) has been sampled four times, between September 2011 and January 2013 under dry and rainy conditions and at different stages of maize growth. This type of soil is assumed to be representative of the whole Laudon’s catchment and of the downstream part of Gabas catchment. Extraction of nitrate from these topsoil samples was performed to characterize their isotopic composition. To do so, soil was dried and crushed above 200 μm, 70 g of soil were added to 140 ml of 0.5M KCl. The mixture was agitated for 2 hours (250 rotations per minute or rpm), centrifuged at 8000 rpm during 35 minutes and filtrated on 0.45 μm nylon membrane. Finally, surface runoff and subsurface drainage have been collected from one ditch and two buried plastic drains (Fig. 2).

All water samples were filtered on 0.45 μm nylon membrane and dispatched into three polyethylene bottles. A fraction (60 ml) of total filtered sample was stored frozen for nitrate and other major anions concentration measurement, another (60 ml) was poisoned with HgCl_2_ (6%) for measurement of isotopic composition of nitrate and a third (250 mL) was acidified to pH = 2 with ultra-pure HNO_3_ for analysis of boron concentration and isotopic ratio but also major cations concentrations. The physical and chemical parameters and concentrations of major anions and cations are available in a supplementary file (Supplementary Table S1).

### Nitrate concentrations, δ^15^N and δ^18^O

For waters and soil extracted samples, nitrate concentrations were analyzed by high-performance liquid chromatography *(HPLC Dionex, AS12 column; Thermo Scientific, Sunnyvale, CA, USA).* δ^15^N and δ^18^O of NO_3_^−^ were determined using the chemical denitrification modified from McIlvin & Altabet^52^ on an isotope ratio mass spectrometer (IRMS, DeltaVplus; Thermo Scientific, Bremen, Germany) in continuous-flow with a purge and trap system coupled with a Finnigan GasBench II system (Thermo Scientific). First step was nitrate reduction to nitrite. Sample, prepared in a salted buffer (NaCl = 0.5 M; pH = 8.5) at 20 μmol NO_3_^−^.L^−1^ passed through a granular activated-cadmium column. Produced NO_2_^−^ was then converted to N_2_O by adding azide (NaN_3_) in a 15 mL solution at 1 μmol NO_2_^−^.L^−1^ sealed in a glass vial. Denitrification reaction was stopped by adding sodium hydroxide to avoid formation of N_2_^53^. The method was calibrated with nitrate standards (USGS-32, δ^15^N = 180‰, δ^18^O = 25.7‰; USGS-34, δ^15^N = −1.8‰, δ^18^O = −27.9‰ and USGS-35 δ^15^N = 2.7‰, δ^18^O = 57.5‰) and verified with an international standard nitrate (IAEA-NO-3, δ^15^N = 4.7‰, δ^18^O = 25.6‰). The precision for δ^15^N was 0.8‰ and 1‰ for δ^18^O (1SD).

Solid samples were air-dried, ground and sealed in tin capsules introduced in an elementar analyzer (Vario PYRO cube, Elementar) where they were oxidized by combustion (at 1120 °C) and reduced into N_2_. δ^15^N of N_2_ was measured with coupled IRMS (IsoPrime, micromass). Calibration was realized with international standards of ammonium sulfate (IAEA-305A, δ^15^N = +39.8 + 0.7‰) and an intern standard (Tyrosine, δ^15^N = +10.01‰). The precision for δ^15^N was 0.3‰ (SD).

### Boron concentrations, δ^11^B and mixing model

#### [*B*] *and δ* ^*11*^ *B analysis*

Samples for B analysis have been selected on the base of nitrate concentrations, δ^15^N and δ^18^O to represent the largest variability. B concentrations were determined on an inductively coupled plasma atomic emission spectroscopy (ICP-OES/AES JY2000). The ^11^B/^10^B isotopic ratios were measured by MC-ICP-MS (Neptune, Thermo Scientific) using a direct-injection nebulizer (d-DIHEN, Analab)^54^. Preliminary B extraction is performed by ion exchange chromatography on Amberlite IRA-743 anionic resin. Retained B was then eluted with HNO_3_ (0.1 and 0.5N)^54^. δ^11^B were measured by sample-standard bracketing with SRM NBS-951 (NIST) boron international standard. Average repeatability of δ^11^B measurements for triplicate analyses of natural water samples was 0.25‰ (2SD)^54^.

#### Mixing model

For each of the Gabas and Laudon river samples (riv), we calculate the proportions (α_x_) of B arising from rain (R), Cretaceous groundwater (GW) and waste-water treatment plants effluents (WWTP) from a set of three mass budget equations on [B]_x_ and δ^11^B_x_:


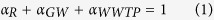



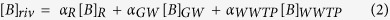



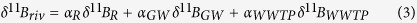


The [B] and δ^11^B values of the mixing end-members were measured in this study (rain and WWTP) or in a previous one (GW)^38^. Rain: [B]_R_ = 1.6 ± 1 ppb and δ^11^B_R_ = 27.7 ± 7‰; Groundwater: [B]_GW_ = 10.5 ± 2 ppb and δ^11^B_GW_ = 25.3 ± 1‰; WWTP: [B]_WWTP_ = 83.3 ± 3 ppb and δ^11^B_WWTP_ = 1.9 ± 2‰;

As discussed in the main text, the mixing doesn’t take into account a possible B input from animal dejections and manure, due to the difficulty of defining an aqueous [B] for this solid end-member. However, its δ^11^B signature (8.6 and −3.3‰) is intermediate between WWTP and river δ^11^B. Thus, the proportion of B arising from WWTP may in fact incorporate animal B input, which cannot be calculated.

### Microbiological Analyses: FRNAPHs and Bacteroidales

Microbiological samples were stored in sterile dedicated flask containing sodium thiosulphate salt (neutralizing agent effective against a wide range of oxidizing substances) used to preserve microorganism. FRNAPHs were enumerated after concentrating 1L of water sample using the membrane filtration-elution method^55^. Infectious FRNAPHs were counted (double agar-layer technique), collected, re-suspended in 1 mL of PBS with 15% glycerol and stored at −20 °C (standard NF EN ISO 10705–1: 2001). Genotyping was performed by one-step real time, reverse transcription polymerase chain reaction (RT-qPCR)^56^. Research of *Bacteroidales* markers (HF183, Rum-2Bac, BacR, BacB2, Pig-1-Bac and Pig-2-Bac) was performed by filtering 1L of sample water through a 0.22 μm pore size polycarbonate membrane. Filter was immersed in a GITC lyses solution and stored at −80 °C. DNA extraction was performed with the Qiamp DNA minikit (Qiagen). Standard curves were calculated for plasmids containing the target sequence. PCR reactions were duplicated for each sample and measurements were performed using a Rotor gene 6000 thermocycler. The results are expressed as a number of copies in 100 mL of water.

## Additional Information

**How to cite this article**: Briand, C. *et al*. Legacy of contaminant N sources to the NO_3_^−^ signature in rivers: a combined isotopic (δ^15^N-NO_3_^-^, δ^18^O-NO_3_^-^, δ^11^B) and microbiological investigation. *Sci. Rep.* **7**, 41703; doi: 10.1038/srep41703 (2017).

**Publisher's note:** Springer Nature remains neutral with regard to jurisdictional claims in published maps and institutional affiliations.

## Supplementary Material

Supplementary Dataset 1

## Figures and Tables

**Table 1:** Hydrological level, chemical, isotopic and microbiological results on Gabas River, Laudon River, surface ditch, buried drains, waste water treatment plant effluents (WWTP) and rain samples.

Sample	Date	Flow	NO_3_^−^	δ^15^N	δ^18^O	B	δ^11^B	Bacteroi-dales	FRNAPHs GII
			mg.L^−1^	‰	‰	μg.L^−1^	‰	*Type*	>*20*%
Laudon	19/10/10	Base	26.2	12.7	7.7	—	—	—	—
Laudon	16/11/10	High	39.3	10.8	5.9	—	—	—	—
Laudon	11/01/11	Base	30.0	12.4	7.6	—	—	—	—
Laudon	15/02/11	Base	23.8	10.6	6.2	11.9	23.3	H C	NO
Laudon	24/02/11	High	—	—	—	—	—	H	NO
Laudon	09/03/11	Base	28.9	11.8	7.6	—	—	—	—
Laudon	05/04/11	Base	19.5	12.9	8.2	—	—	H	NO
Laudon	27/05/11	Base	20.9	12.0	7.0	—	—	—	—
Laudon	21/06/11	Base	23.6	12.7	8.2	—	—	—	—
Laudon	12/08/11	Base	26.6	12.6	8.4	1.8	13.5	—	—
Laudon	06/09/11	Base	14.3	13.4	9.3	—	—	—	—
Laudon	19/09/11	Base	15.8	12.8	8.9	13.9	25.2	C	NO
Laudon	05/01/12	High	35.1	11.4	6.4	—	—	H	NO
Laudon	06/01/12	Base	20.1	11.6	6.2	—	—	HC	YES
Laudon	19/01/12	Base	32.1	11.4	6.2	12.1	24.0	—	—
Laudon	21/03/12	Base	18.1	13.8	8.9	—	—	HC	*
Laudon	30/04/12	Base	12.0	9.8	6.2	9.2	24.2	x	—
Laudon	20/06/12	Bas	23.8	14.0	9.3	—	—	x	—
Laudon	21/08/12	Base	28.7	11.0	7.6	6.5	26.8	C	*
Laudon	23/10/12	Base	30.3	11.1	7.6	—	—	—	—
Laudon	15/01/13 16:00	High	—	—	—	—	—	H C P D	NO
Laudon	16/01/13 11:00	High	17.4	8.8	5.5	12.1	23.7	H C P D	NO
Laudon	16/01/13 16:00	High	28.2	8.0	5.4	—	—	—	—
Laudon	17/01/13 11:00	High	—	—	—	—	—	H C P D	NO
Laudon	17/01/13 15:00	High	36.3	9.1	5.6	—	—	H C P D	YES
Laudon	18/01/13	High	—	—	—	—	—	H C P D	NO

Gabas	16/11/10	High	37.2	11.6	5.9	—	—	—	—
Gabas	15/02/11	Base	20.4	12.4	9.1	7.5	21.2	C	NO
Gabas	24/02/11	High			—	—	—	H C	NO
Gabas	05/04/11	Base	18.1	13.3	7.8	—	—	H	NO
Gabas	12/08/11	Base	13.6	14.6	8.0	6.8	20.7	—	—
Gabas	06/09/11	Base	38.2	11.7	8.2	—	—	—	—
Gabas	05/01/12	High	45.4	8.6	3.8	6.1	23.5	H	YES
Gabas	06/01/12	Base	—	—	—	—	—	H C P	YES
Gabas	19/01/12	Base	34.8	11.7	7.2	—	—	—	—
Gabas	21/03/12	Base	18.2	13.5	8.4	—	—	H	YES
Gabas	30/04/12	Base	23.1	11.0	5.8	7.6	23.9	H C	*
Gabas	21/08/12	Base	19.8	12.0	7.4	4.4	26.3	H	*
Gabas	16/01/13 11:00	High	26.8	10.2	6.1	9.0	23.1	H C P D	YES
Gabas	17/01/13 11:00	High		—	—	—	—	H C P D	NO
Gabas	18/01/13	High	—	—	—	—	—	H C P D	YES

Ditch 1	19/01/12	Base	35.7	11.6	7.5	10.5	22.0	H	NO
Ditch 1	20/03/12	Base	20.5	11.5	7.4	13.1	17.6	H	NO
Ditch 1	30/04/12	High	18.0	10.5	5.1	10.5	18.8	H	YES
Ditch 1	17/01/13	High	40.1	9.7	7.0	10.9	17.1	H	YES

Drain 1	19/01/12	Base	56.9	19.6	11.0	7.3	26.7	—	—
Drain 2	19/01/12	Base	39.3	12.9	7.9	50.3	16.1	—	—
Drain 2	15/01/13	High	63.4	11.4	7.3	37.0	11.5	—	—

WWTP 1 effluent	19/01/12	Base	86.4	17.3	8.5	83.3	1.9	H	NO
WWTP 1 effluent	15/01/13	High	58.0	10.0	6.4	—	—	—	—
WWTP 2 effluent	19/01/12	Base	28.2	12.6	7.9	—	—	—	—

Rain	20/03/12	Base	2.7	1.6	78.6	1.6	27.7	—	—

Cretaceous Aquifer	19/10/10–17/01/13	—	23.3 ± 1.5	8.2 ± 1.2	5.5 ± 0.8	9.8 ± 2.7	24.9 ± 1.5	—	—

Bacteroidales measured at significant concentrations (>10^4^ copies/100 mL) with H: human, C: cattle, P: pig, D: duck and FRNAPHs of GII, YES: representing more than 20% of total phages on more than 12 phages (number of plaque forming units available for typing by Petri dish), NO: GII detected <20% or <12 phages, x: under significant limit, ^*^ under quantifying limit.

**Table 2:** Chemical and isotopic data measured for soil extracted nitrates and solid samples of chemical fertilizers (urea), poultry fresh dejection and manure.

Solid Samples	Date	NO_3_^−^	δ^15^N	δ^18^O	B	δ^11^B
		mg.g^−1^	‰	‰	μg.g^−1^	‰
Soil Extraction	27/09/2011	52	4.2	9.2	—	—
Soil Extraction	14/12/2011	17	0.8	9.3	—	—
Soil Extraction	24/08/2012	23	−2.5	11.4	—	—
Soil Extraction	18/01/2013	16	−11.8	5.5	—	—
Urea	—	—	0.9	—	<LD	*
Poultry Manure	—	—	9.3	—	5	8.6
Poultry Fresh Dejection	—	—	3.6	—	23	−3.3
